# Optimizing workflow efficiency for analyzing low molecular weight endogenous peptides in colostrum†

**DOI:** 10.1039/d4ra03199g

**Published:** 2024-09-16

**Authors:** Priti Panchal, Reeju Rani, Rohit Kumar, Seema Malik, Manishi Mukesh, Jai Kumar Kaushik, Monika Sodhi, Ashok Kumar Mohanty, Sudarshan Kumar

**Affiliations:** a ICAR-National Dairy Research Institute, Proteomics and Cell Biology Lab, Animal Biotechnology Division (ABTD) Karnal Haryana 132001 India kumarsudershan@gmail.com; b College of Public Health and Human Science, Oregon State University Corvalis OR 97331 USA; c ICAR-National Bureau of Animal Genetic Resources Karnal Haryana 132001 India; d ICAR-Central Institute for Research on Cattle Meerut India

## Abstract

Bovine milk and colostrum play pivotal roles in the nutritional support of both human and bovine infants. Colostrum, the initial milk secretion, is crucial for neonatal growth, providing essential nutrients, growth factors, immunity, and defense mechanisms through a diverse array of bioactive compounds, including bioactive proteins and peptides. Peptidomics, leveraging the potential health benefits of peptides derived from food and body fluids, has become prominent in contemporary research. Endogenous peptides (EPs) have gained notable scientific and commercial interest due to their potential biofunctional significance in areas such as immune health, antimicrobial, anti-inflammatory, antihypertensive, and antioxidative studies. In this investigation, we aimed to extract and analyze low molecular weight EPs from colostrum using four distinct peptide extraction methods, previously employed for EPs extraction from other bodily fluids. The efficiency of these methods was systematically compared and analysed to identify the most effective extraction technique for maximizing the identification of low molecular weight EPs from colostrum. This study represents a pioneering effort as no prior research has systematically compared different extraction methods for low molecular weight EPs from colostrum. Given the unique physical and chemical composition of colostrum compared to milk and other body fluids, a comprehensive analysis of EPs extraction methods was deemed essential. In the present study, we successfully extracted over 3200 EPs from colostrum using trichloroacetic acid (TCA) and a molecular weight cut off (MWCO) extraction method. The findings of this study revealed the extraction of EPs from colostrum, demonstrating potential inherent bioactivities as predicted by *in silico* tools.

## Abstract 2

A novel approach to extract EPs from colostrum using peptide extraction methods combined with ultracentrifugation was developed. Over 3200 EPs were extracted from bovine colostrum enabling the identification of less abundant proteins and peptides.

## Graphical abstract

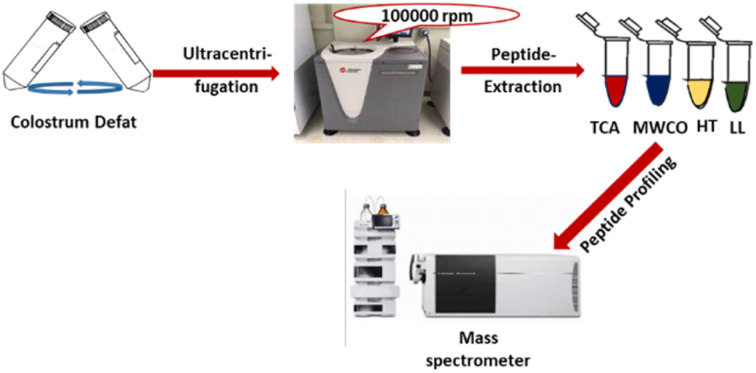

## Introduction

Peptidomics is the branch of proteomics that refers to the qualitative and quantitative analysis and characterization of the entire pool of peptides existing in the biological samples.^1–4^ Peptidomics is a complex field that has successfully been applied in several areas of research for quantitation and identification of food bioactive peptides, characterization of food processing related proteolysis, profiling of human milk peptides, profiling of peptides in gastric samples *etc.* Despite the thorough investigation of peptides produced by hydrolysis or fermentation, native peptides already existing in the mammary gland or other parts of the body are usually neglected. EPs are naturally existing peptides in the biological fluids. These EPs emanate by translational or proteolytic degradation through the action of proteases naturally present in the same biological system.^1^ EPs are the low molecular weight components typically consisting of 2–50 amino acids (AA). EPs are extensively distributed throughout almost all biological fluids including blood plasma,^1,5^ urine,^6^ colostrum,^7,8^ milk^9^ and milk products^10–18^ and these peptides have several important biological functions. Peptidome profiling of 10 commercial dairy products identified 66 unique bioactive peptides with immunomodulatory, anti-hypertensive, anti-thrombotic, anti-microbial, anti-oxidative and opioid agonist functions.^18^ EPs get directly absorbed in the intestine and provide potential health benefits to new-borns. In a recent study, 8393 EPs were identified and quantified in the milk of two different breeds of camel. The identified peptides were functionally annotated to determine the bioactive functionality.^10^ Recently, a detailed identification and characterization of short EPs in milk and its by-products including whole whey, skimmed whey, and whey permeate were carried out with the help of high-resolution mass spectrometry, and in total 79 short EPs were identified.^11^ Several studies have been performed in recent years for the peptidome analysis of EPs in human, bovine, camel and other species milk.^10,17,19–23^ Though milk has been explored widely for the peptidome studies, comparatively less number of studies are available on the investigation of colostrum derived peptides. Several studies have proven the immunological and biological significance of colostrum^24,25^ especially in the neonate health development,^25^ immunomodulation^26,27^, regulation of cytokine production and improvement of gut health.^28^ Colostrum contains a wide array of immune boosting factors including antimicrobial,^26,29^ antioxidative,^30^ anti-inflammatory, antiviral and growth promoting proteins and peptides, immunoglobulins, lactoferrin, insulin like growth factor and other. A recent study reported isolation of bioactive peptides from buffalo colostrum whey produced by bacterial fermentation. It identified 40 potential immunomodulatory sequences and 16 antimicrobial sequences.^26^ And most of the biologically important components are at higher concentration in colostrum than in milk.^31^ Nissen *et al.* performed a comparative study between bovine colostrum proteome and milk proteome to rank the proteins mutually and to generate a ratio between colostrum and milk proteome. They observed the highest concentration of most of proteins including osteopontin, total immunoglobulins, lactotransferrin, apolipoprotein, milk amyloid protein A, haptoglobin, transforming growth factor β-2 and transforming growth factor β-1 in the colostrum. The concentration of these proteins declined greatly in the milk.^24^ Colostrum was found to have developmental and immunological importance since several proteins such as immunoglobulins, thyroglobulins, RNA binding proteins were at higher level in colostrum than in milk of Mediterranean and Murrah buffalo.^25^ Colostrum is also found to promote the performance of athlete in post exercise inflammatory responses by reducing the hyper-permeability and maintaining the junctional integrity of the intestine.^32^ Colostrum derived peptides have recently been explored since these peptides are found to have significant bioactive functionality. Recently, a group of scientists studied the colostrum derived peptides generated by simulated digestion in oral, gastric, and duodenal phases. Some of the identified peptide sequences showed potential antimicrobial activity when predicted bioinformatically.^33^ Ashok and coworkers investigated the antioxidative potency of peptic hydrolysate of buffalo colostrum whey and observed that the hydrolysates diminished the increased level of ROS, H_2_O_2_ and catalase.^30^ While most of previous studies focused on the peptides generated by *in vitro* digestion of the proteins, there are very few studies on the profiling or identification of colostrum-derived EPs. In a previous study three naturally present peptides named isracidin, casecidin 15 and 17 were isolated and characterized from bovine colostrum and were found to have antimicrobial activity against *E. coli*.^34^ Few other studies on colostrum-derived EPs prove the significance of these peptides.^7,35,36^ Given the different numbers of EPs reported in different studies, a comprehensive research is required to achieve an effective extraction method of EPs from the colostrum in order to form an accurate and reproducible peptidome base. So, in this study, we aimed to explore and compare the efficiency of various methods which has been used previously for EPs extraction form various body fluids but colostrum. We introduced modifications in these methods in order to achieve the maximum identification of colostral peptides.

## Material and method

### Sample collection

Bovine colostrum samples were collected within 24 h of calving in sterile tubes and stored at −80 °C until further use to minimize the milk protease activity, which has the potential to modify the peptide profile.

### Ultracentrifugation

**Fig. 1:**
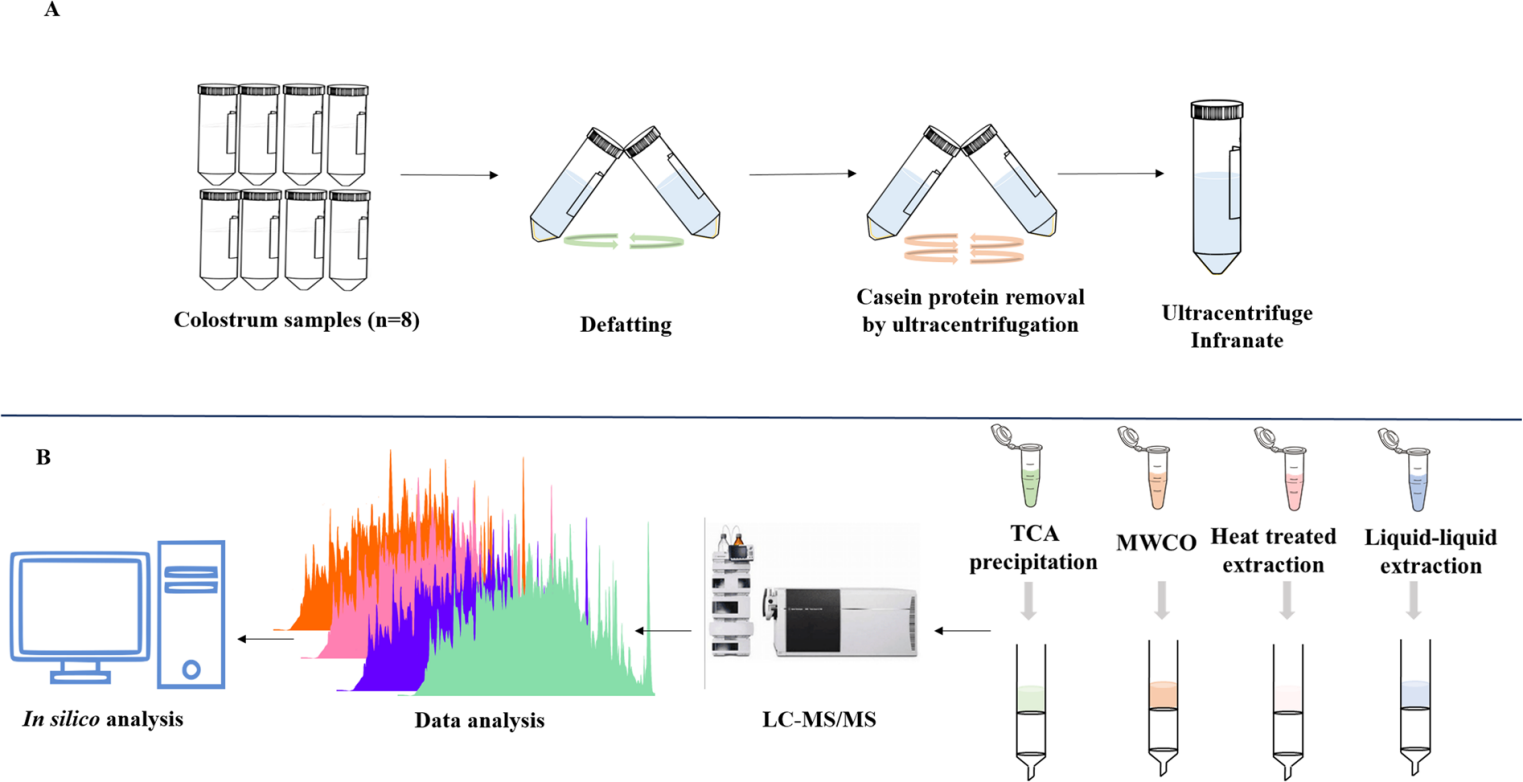
Flow diagram depicting the collection and processing of colostrum samples. (A) After collection, colostrum was defatted and ultracentrifuged at high speed and infranate was collected. (B) For the extraction of EPs, four different peptide extraction methods were applied: TCA precipitation, MWCO, heat treated and liquid–liquid extraction. The samples were desalted and analysed using mass spectrometry.

The fresh colostrum samples were pooled (*n* = 8) and defatted by centrifugation at 1000 × *g* for 20 min at 4 °C. Fat layer was removed and the skimmed colostrum infranate was collected in fresh tubes and stored at refrigerated condition for further use. Skimmed colostrum infranate was then subjected to ultracentrifugation in order to deplete casein which accounts for about 80% of the total proteins. Due to the abundance of casein in the colostrum, other less abundant proteins and peptides would be difficult to identify. For this skimmed colostrum was centrifuged at 70 000 × *g* for 60 min at 4 °C and then the remaining fat layers was removed, supernatant was collected and again centrifuged at 100 000 × *g* for another 60 min at 4 °C. Collected supernatant (casein-depleted colostrum) was stored at −80 °C till further use (Fig. 1A).

### Peptide extraction

Peptide extraction from colostrum samples was performed by four different extraction methods. Equal volume of ultracentrifuged colostrum was taken for further processing (Fig. 1B).

### Molecular weight cutoff

Low molecular weight EPs were isolated through centrifugation-based filtration utilizing a 9 kDa molecular weight cutoff (MWCO) device (Protein Concentrators®, Thermo Scientific, Pierce Biotechnologies, Rockford, USA). Initially, the filter underwent a washing step with ultrapure water, employing centrifugation at 3000 × *g* for 15 minutes at 4 °C. Subsequently, casein-depleted colostrum was mixed with acetonitrile (ACN) (Honeywell, France) at a 2 : 1 ratio in an ACN-compatible tube. After vortexing for 5 min and a 30 minute incubation at 4 °C, the tube's contents were transferred into the filtration device and subjected to centrifugation under above mentioned conditions for 20 minutes. The retentate, harboring proteins of higher molecular weight, was discarded, while the filtrate containing peptides was collected, stored at −20 °C and subjected to peptide enrichment and desalting using spin columns.

### TCA precipitation

EPs were extracted using the protocol established by Ferranti *et al.*^2^ In summary, casein-depleted colostrum was treated with an equal volume of 20% trichloroacetic acid (TCA) (SRL, India) solution (w/v) in water, resulting in a final TCA concentration of 100 g L^−1^. The mixture was vigorously vortexed for 5 minutes and subsequently subjected to centrifugation at 3000 × *g* for 20 minutes at 4 °C. The resulting supernatant was carefully collected and then processed through peptide enrichment and desalting.

### Liquid–liquid extraction

This approach was adapted from established procedures with some modifications. To isolate EPs from colostrum, 2400 μL of methanol (Honeywell, France) was mixed with 800 μL of casein-depleted colostrum and thoroughly vortexed. Following this, 2135 μL of chloroform (SRL, India) was added and the mixture was vortexed once again. The mixture was continuously shaked for 1 hour at 4 °C. Subsequently, 1065 μL of cold water was added, and the sample underwent centrifugation at 10 000 × *g* for 20 minutes at 4 °C. The organic phase was discarded, and the remaining aqueous phase was collected followed by concentration in a speed vac concentrator (Eppendorf Concentrator Plus™, Eppendorf, Hamburg, Germany). The concentrate was then reconstituted in ultrapure water and subjected to peptide enrichment and desalting.

### Heat-treated extraction

In this methodology, casein-depleted colostrum was heated in a microwave for a duration of 30 seconds. Subsequently, the resulting liquid fraction was collected and subjected to centrifugation at 10 000 × *g* for a duration of 20 minutes at 4 °C. The supernatant, thus obtained, underwent collection and filtration through a molecular weight cutoff filter with a 10 kDa molecular weight cutoff. The resultant filtrate was further processed through peptide enrichment and desalting using spin desalting columns.

### Peptide enrichment and desalting

Prior to the identification of peptides through LC-MS/MS, it is imperative to undertake the purification and enrichment of peptides. This is essential to enhance sensitivity in peptide identification and address the challenge of signal suppression arising from the concurrent presence of non-target molecules. The extracted peptides were subsequently subjected to desalting using solid-phase extraction. Samples were desalted using Pierce™ Peptide Desalting Spin Columns (Thermo Scientific, Rockford, USA). Column was first activated using 100% methanol followed by equilibration with 2% ACN, 0.1% TFA. The sample was loaded onto the column, followed by column wash with 2% ACN, 0.1% TFA and the bound peptides were subsequently eluted from column by 40% ACN, 0.1% TFA. The eluate was dried down by vacuum concentrator (Eppendorf Concentrator Plus™, Eppendorf, Hamburg, Germany) and identification of peptides was achieved by mass spectrometry.

### Mass spectrometry

The identification of colostrum-derived EPs was done using high-resolution mass spectrometry (HRMS) (Agilent Technologies, Santa Clara, CA, USA) at the National Chemical Laboratory (Venture Center), Pune. All the samples were reconstituted in 50 μL 0.1% formic acid. The injection volume was 25 μL and column used was Zorbax Eclipse Plus c18 column (150 mm × 2.1 mm, 1.8 μm). Sample was injected at a flow rate of 0.5 mL min^−1^. MS analysis was performed with Agilent 6550 UHD QTOF MS equipped with Dual AJS ESI ion source. The chromatographic gradients were as shown below A: water with 0.1% formic acid; B: 0.1% formic acid in 90% ACN. The gradient employed was ramped from 0 to 8% B for 0 to 5 min, 8 to 27% B from 5 to 24 min, 27 to 99% B from 24 to 55 min, followed by 99% B for 5 min. The acquisition mode applied was auto MS/MS and precursor ion formed were acquired in the range of 100–1700 *m*/*z* in positive polarity. The scan rate for MS was 2 spectra per second and for MS/MS it was 3 spectra per second. Other instrument parameters used were: gas temp – 270 °C, gas flow – 11 L min^−1^, Nebulizer – 25 psig, sheath gas temp – 295 °C, sheath gas flow 10.

### Data analysis

Raw data file was analysed through Trans Proteomic Pipeline version 6.3.0 Arcus as described by Kumar *et al.*, 2021 (ref. 6) with some modifications. Briefly, .d file were converted to mzML format with the help of MSconvert (a proteowizard tool). This newly generated mzML file was then searched using comet search parameters for MS/MS search against UniProt *Bos taurus* proteome database (Proteome Id UP000009136). The search was performed using parameters for undigested peptides and for N and C-terminal unspecific cleavage. Up to four potential variable modifications were allowed on each peptide including phosphorylation at serine, threonine and tyrosine; methionine oxidation; asparagine and glutamine deamidation. Peptide mass tolerance was set 20 ppm and maximum missed cleavage were allowed up to 2. Remaining parameters were set as default. The .xml file generated after comet search was subjected to peptide prophet and iProphet. Peptide prophet and iProphet were run in Xpress in label free mode to calculate the peak area. Percentage peak area occupied by the sequences was calculated by dividing the sum of peak area to the peak area occupied by individual sequence. Finally, protein prophet was used to assign a protein probability score to each identified peptide. High confidence peptides were accepted based on the iProphet probability cut-off and error rate. An error rate of <0.05 was accepted for identification of the peptides as correct hit. Peak area of the identified sequences was calculated by running the peptide prophet and iProphet in label free mode.

### Statistical analysis

The qualitative analysis of identified peptides was conducted utilizing Microsoft Excel. Venn diagrams were constructed employing Venny 2.1.0.^37^ Bar charts were generated using both MS Excel and GraphPad Prism version 8.0.1 (Graph Pad Software Inc., San Diego, CA, United States). Additional plots were crafted using SRplot,^38^ and BoxplotR.^39^ The molecular weight of identified peptides was computed through the utilization of TumorHPD.^40^ To assess the hydropathicity of peptide sequences, the GRAVY calculator (https://www.gravy-calculator.de) was employed.

## Results

### Evaluation of number of identified peptides in different methods

**Fig. 2:**
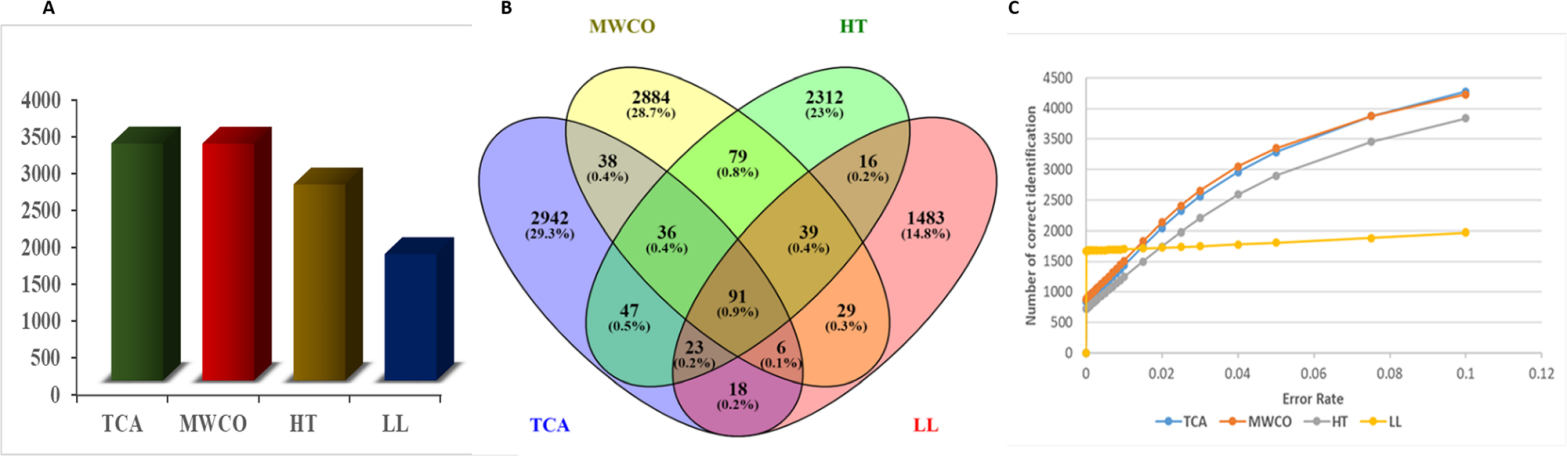
Peptide sequences identified across different extraction methods. (A) Frequency of identified sequences in four methods: TCA precipitation, MWCO, HT and LL extraction methods. (B) Venn diagram displaying the number of common peptide sequences among TCA, MWCO, HT and LL methods. (C) Number of correct identification against the error rate in each method.

Total number of unique peptides identified in Yak colostrum are shown in Fig. 2A. The total number of unique peptides extracted were 3211, 2656, 3210 and 1716 in TCA precipitation, heat-treated, MWCO and Liquid–Liquid (LL) extraction, respectively. Peptide sequences were selected with an error rate of <0.05 (Fig. 2C). TCA precipitation and MWCO extraction methods were found to be more effective for extraction of low molecular weight EPs from Yak colostrum since both the methods identified equal number of peptides. TCA precipitation and MWCO methods yielded highest number of peptides among all the methods. Venn Diagram for the number of common sequences identified in the three methods shows that only 91 sequences were common through all the methods *i.e.* TCA precipitation, MWCO method, heat treated and LL extraction (Fig. 2B). A total of 171 sequences were common between TCA precipitation and MWCO method. And 197 sequences were common among TCA and heat treated extraction method while 138 sequences were common in TCA and LL extraction method. 2941, 2883, 2311, 1482 sequences were exclusively identified in TCA, MWCO, HT and LL extraction methods respectively.

### Evaluation of amino acid profile of identified peptides in different methods

**Fig. 3:**
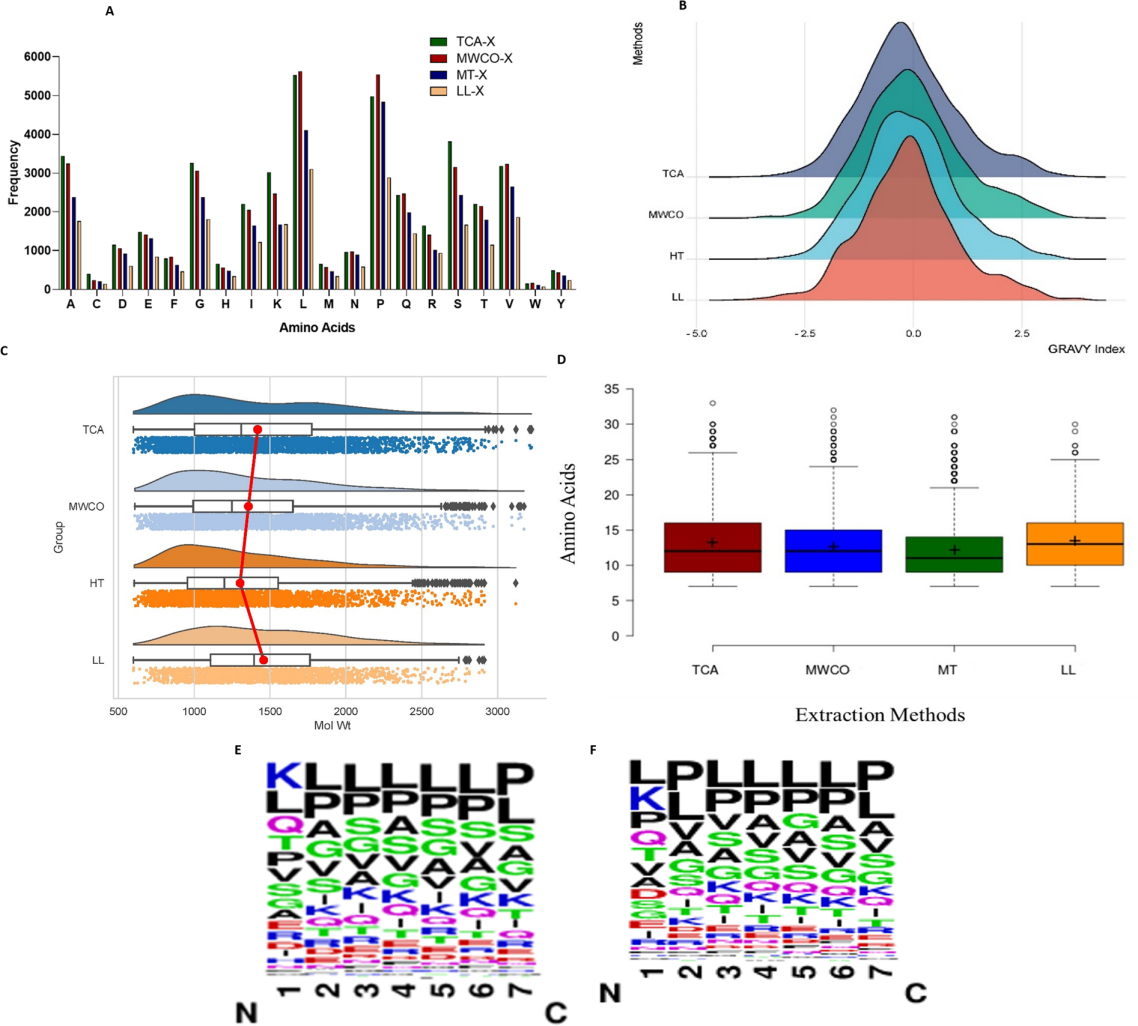
Physico-chemical characterisation of identified sequences. (A) Amino acid frequency of identified sequences across four methods. (B) Gravy index of sequences. (C) Molecular mass of identified sequences in each method. (D) Peptide sequence length (in AA) of identified sequences across all four methods. (E) Sequence logos (7 amino acid from N-terminus) of sequences identified in TCA and (F) in MWCO.

We summarized information regarding the prevalence of amino acids in the identified sequences as depicted in Fig. 3A. Leucine emerged as the most widely distributed amino acid in peptide sequences obtained through all extraction methods, except in LL where proline prevailed as the most abundant. Hydrophobic amino acids, such as P, G, L, V, and A, were consistently present in high frequency across all extraction methods. Additionally, hydrophilic amino acids like S, T, and Q were also enriched abundantly in all four methods.

Further, analysis of the sequence logos for the first seven amino acids from the N-terminus of sequences identified *via* the TCA and MWCO methods was performed (Fig. 3E and F). It was observed that the first position was predominantly occupied by lysine, followed by leucine and glutamine in TCA method. While for the MWCO method, leucine, lysine, and proline were the most common in the first position. From the second position onwards, leucine and proline were the most abundant amino acids in both methods.

### Evaluation of mass range and sequence length of identified peptides

The graphical representation in Fig. 3C illustrates that all employed methods primarily resolved low molecular weight peptides within the range of 700 Da to <3300 Da. Notably, LL peptide extraction method exhibited an absence of peptides exceeding 3000 Da. Across all methods, the prevalence of detected peptides fell within the range of 700 Da to 2000 Da. Further analysis revealed that TCA, MWCO and heat treated extraction methods predominantly identified peptides in the range of 900 Da to <1000 Da (TCA – 10.64%, MWCO – 10.68%, HT – 12.01%), while LL extraction detected the highest number of peptides within the 1100 Da to <1200 Da range (10.31%) despite method-specific variations, the majority of peptides obtained through the different extraction methods displayed comparable patterns, both in terms of the number of amino acid residues and the distribution of molecular weights.

Further, it was observed that all the applied methods consistently retrieved sequences spanning from 7 amino acids (AA) to 30 amino acids (Fig. 3D). Significantly, the predominant sequence length ranged from 7AA to 18AA with 85.23%, 89.47%, 87.34%, and 91.41% for TCA, MWCO, LL extraction, and heat-treated extraction, respectively. It is noteworthy that the amino acid sequences obtained through MWCO and heat-treated extraction were significantly shorter, respectively accounting for 50.90% and 55.15%, within the range of 8AA to 12AA.

### Hydropathicity of identified peptides

To determine the hydrophobicity of the sequences, we computed GRAVY index values for sequences identified through all four methods (Fig. 3B). As depicted in the figure, peptides identified across these methods predominantly exhibited GRAVY values ranging from −0.25 to 0.25. Nevertheless, 54–58% of the peptides identified through these methods displayed GRAVY index values below 0, signifying hydrophilic characteristics. Concurrently, there was also a presence of hydrophobic peptides, constituting 41–45% across different methods.

While each method resulted in identification of both hydrophilic and hydrophobic peptides, the prevalence of hydrophilic sequences was notably prominent. TCA extraction method exhibited enrichment of hydrophilic peptides, constituting 58.17%, surpassing the other three methods (MWCO – 54.26%, HT – 54.94%, LL – 56.36%).

### Parent protein of identified EPs

**Fig. 4:**
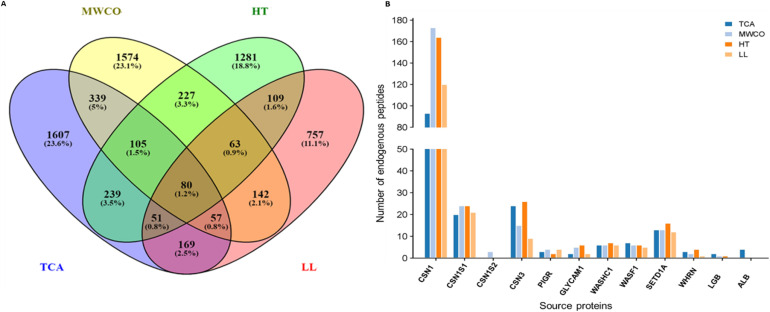
Parent proteins analysis of identified endogenous peptides. (A) Venn diagram displaying the number of parent proteins from which the endogenous peptides were originated among four different methods; TCA, MWCO, HT, LL. (B) Graph showing number of identified peptides from major milk proteins. CSN1 – beta casein; CSN1S1 – alpha S1 casein; CSN1S1 – alpha S2 casein; CSN3 – kappa casein; PIGR – polymeric immunoglobulin receptor; GLYCAM1 – glycosylation dependent cell adhesion molecule 1; WASHC1 – WASH complex subunit 1; WASF1 – wiskott-aldrich syndrome protein family member 1; SETD1A – histone-lysine *N*-methyltransferase; WHRN – whirlin; LGB – beta lactoglobulin; ALB – serum albumin.

Parent proteins of identified EPs were identified and analysed as shown in Fig. 4. All the peptides identified through TCA, MWCO, HT, LL extraction methods were derived from 2647, 2587, 2155 and 1428 proteins respectively (Fig. 4A). Remarkably, 80 parent proteins were consistently identified across all the methods with TCA and MWCO exhibiting the highest commonality, sharing 581 proteins, accounting for 12.5%, as depicted in the Fig. 4A. This huge number of unique protein across the methods is due to different members of a family or groups have different Uniprot IDs.

### Functional annotation of identified sequences

**Fig. 5:**
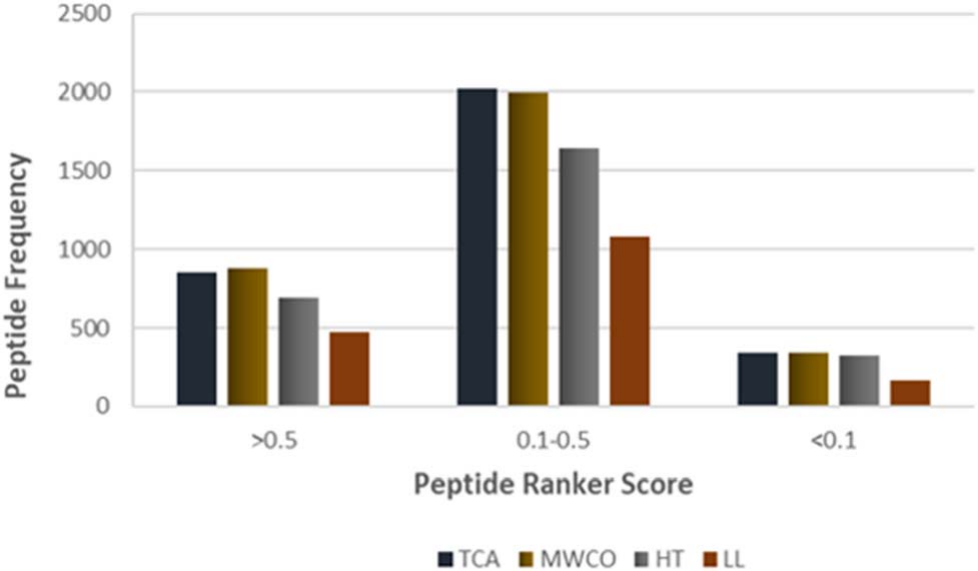
PeptideRanker score of identified sequences against the frequency of peptides identified across TCA, MWCO, HT and LL extraction methods.

Identified peptide sequences were scrutinized for potential bioactive attributes utilizing bioinformatics tools. Peptide bioactivity prediction was executed employing PeptideRanker. PeptideRanker is neural network based tool which assigns a bioactive probability to each peptide sequence, ranging from 0 to 1, where scores nearing 1 indicate a high confidence for bioactive potential of given peptide sequence. Peptides attaining a score >0.50 were deemed to possess a significant probability to exhibit bioactivity. Remarkably, a substantial proportion of the identified sequences, exceeding 25%, exhibited scores greater than 0.5 in PeptideRanker, indicating a high-confidence bioactivity prediction (Fig. 5). Furthermore, over 60% of the sequences were within the intermediate range of PeptideRanker scores, ranging from 0.1 to 0.5, suggesting a moderate probability of bioactivity. These findings collectively underscore the potential bioactivity inherent in the identified sequences across employed methods.

Peptides sequences were screened in Milk Bioactive Peptide Database (MBPDB) to analyse bioactivities already reported elsewhere. Table 1 below shows MBPDB database analysis results of peptide sequences extracted through all the applied methods.

**Table 1:** Table reporting the bioactive functionality of identified sequences retrieved from MBPDB and peptide extracted from the extraction method used in this study

Peptide sequence	Protein name	Protein ID	Species	Bioacivity	Method			
					TCA	MWCO	HT	LL
KVLPVPQK	P02666	Beta-casein	Bos taurus	Antioxidant, immunomodulatory, anti-inflammatory	✓	✓	✓	✓
EPVLGPVRGP	P02666	Beta-casein	Bos taurus	Cytomodulatory	✓	✓	✓	✓
VLGPVRGPFP	P02666	Beta-casein	Bos taurus	ACE-inhibitory	✓	✓	✓	✓
EPVLGPVRGPFP	P02666	Beta-casein	Bos taurus	ACE-inhibitory	✓	✓	✓	✓
SQSKVLPVPQKAVPYPQ	P02666	Beta-casein	Bos taurus	Antioxidant	✓	✓	✓	✓
TPVVVPPFL	A0A344 × 7B9	Beta-casein	Bos mutus	Anticancer	✓	✓	✓	✓
KVLPVPQ	P02666	Beta-casein	Bos taurus	ACE-inhibitory, immunomodulatory, anti-inflammatory	✓	✓	✓	✓
RPKHPIKHQ	P02662	Alpha-S1-casein	Bos taurus	ACE-inhibitory	✓	✓	✓	
RPKHPIK	P02662	Alpha-S1-casein	Bos taurus	Antimicrobial	✓		✓	
MPFPKYPVEP	P02666	Beta-casein	Bos taurus	ACE-inhibitory	✓		✓	
ELNVPGEIVES	P02666	Beta-casein	Bos taurus	Antimicrobial	✓		✓	✓
SQSKVLPVPQ	P02666	Beta-casein	Bos taurus	ACE-inhibitory	✓		✓	✓
SKVLPVPQ	P02666	Beta-casein	Bos taurus	ACE-inhibitory	✓			
LPQNIPPLT	P02666	Beta-casein	Bos taurus	DPP-IV inhibitory		✓	✓	
NLHLPLPLL	P02666	Beta-casein	Bos taurus	ACE-inhibitory		✓	✓	
VPYPQRDMPIQAFL	P02666	Beta-casein	Bos taurus	Antimicrobial		✓	✓	
YPFPGPIPNS	P02666	Beta-casein	Bos taurus	Antianxiety		✓	✓	
LVYPFPGPIPNSLPQN	P02666			ACE-inhibitory		✓	✓	
YQEPVLGPVRGPFPIIV	P02666	Beta-casein	Bos taurus	ACE-inhibitory, anticancer, antimicrobial, antithrombotic, immunomodulatory		✓	✓	✓
LYQEPVLGPVRGPFPIIV	P02666	Beta-casein	Bos taurus	Immunomodulatory		✓	✓	✓
LLYQEPVLGPVRGPFPIIV	P02666	Beta-casein	Bos taurus	ACE-inhibitory		✓	✓	✓
TPVVVPPFLQP	P02666	Beta-casein	Bos taurus	ACE-inhibitory		✓		✓
QEPVLGPVRGPFPIIV	P02666	Beta-casein	Bos taurus	ACE-inhibitory		✓		✓
YQEPVLGPVR	P02666	Beta-casein	Bos taurus	ACE-inhibitory, antioxidant, antithrombotic, Immunomodulatory		✓		
ENLHLPLPLL	P02666	Beta-casein	Bos taurus	ACE-inhibitory		✓		
EPVLGPVRGPFPIIV	P02666	Beta-casein	Bos taurus	ACE-inhibitory			✓	
VENLHLPLPLL	P02666	Beta-casein	Bos taurus	ACE-inhibitory			✓	
DVENLHLPLPL	P02666	Beta-casein	Bos taurus	Antimicrobial				✓

## Discussion

We realized that this is not the first study to compare the methods for EPs extraction. Recently, Dingess *et al.*^41^ compared different methods for extraction of EPs in human milk. In another study, four different protocols were compared for the extraction and purification of short endogenous plasma peptides.^5^ In a similar study EPs extraction method including ACN precipitation, ultrafiltration and size exclusion chromatography were evaluated from human plasma.^1^ All of the previous studies investigating EPs extraction were conducted on either human or bovine milk or plasma or urine.^13,42^ Each biological fluid including milk, colostrum or serum is quite different in the composition as well as physical properties. A study conducted on the physical properties of colostrum and milk observed the decreased concentration of casein and IgG, change in colour from yellow to white, change in pH as the colostrum transits into milk.^31^ Furthermore, the peptidome profiling of colostrum poses significant challenges due to its high viscosity compared to mature milk. This viscosity, along with other factors, hinders the development of a reliable methodology for extracting low molecular weight EPs. The major technical hurdle in the extraction and analysis of low molecular weight EPs is that the majority of the colostrum proteome and peptidome is covered by the abundant proteins and the peptides present in the sample. These dominant proteins and peptides suppress the signal of low-abundant and low molecular weight peptides during MS analysis and consequently, hampers the sensitivity of detection. So, peptide extraction workflow adapted for milk, serum or urine cannot be directly applied to colostrum making it necessary to explore the efficiency of these methods on colostrum. In a previous study, Ning *et al.* extracted EPs from colostrum using TCA precipitation method and for this, the colostrum was diluted fourfold with ultrapure water. Although over 10 000 peptides were identified in this study, they originated from approximately 1100 unique parent proteins.^43^ In the present study, although the number of peptides identified was 3211, the source proteins of these peptides were over 2500. Interestingly, 47.82% of EPs in Ning *et al.*'s study were from casein, while in the present study, casein accounted for only 4.26% in the TCA method and 6.69% in the MWCO method. This difference is likely due to the enhanced identification of peptides from low-abundance proteins. This suggests that while diluting colostrum effectively reduces its viscosity, it does not adequately address the challenge of detecting low-abundance proteins, which remain masked by highly-abundant proteins. Consequently, the sensitivity for detecting low-abundance proteins and peptides is compromised due to the presence of dominant proteins and peptides.

To achieve comprehensive peptidome and proteome coverage, combining ultracentrifugation with peptide extraction methods, is more effective for colostrum. This approach is supported by previous studies demonstrating that extensive proteome coverage, particularly for low-abundance proteins, was achieved in colostrum through ultracentrifugation. High-speed centrifugation was shown to significantly enhance the detection of low-abundance proteins, thereby contributing to a more exhaustive proteomic analysis.^44^

Another bottleneck in the investigation of the low molecular weight EPs is that EPs are submerged in complex biological fluid which contains wide variety of compounds including lipids, carbohydrates, fats and salts. Also, EPs have huge heterogeneity in their size, abundance, charge and chemical composition. All these factors influence the extraction as well as detection of peptides. Therefore, it requires a unique workflow for the extraction of peptides.^4,45^

Very few studies have examined the EPs profile of colostrum, and all of these studies are based on any particular method for the extraction of EPs. Laboratory and pilot scale peptide extraction was performed using ultrafiltration method for comprehensive peptide analysis from the whey permeate. They identified a total of 212 and 238 unique peptides in both the methods respectively and all of the peptides were predominantly casein derived.^8^ In another study Jorgensen and co-workers isolated putative bioactive peptides from nondigested colostrum and their immune modulation response was assessed against some bacterial ligands such as lipopolysaccharides and peptidoglycans.^7^

The implementation of proficient and specialized sample preparation is imperative for the successful conduct of peptidomic studies. Unlike tryptic peptides, EPs lack specific C-terminal residues such as K and R, complicating their analysis. Therefore, developing an effective method for their efficient extraction is necessary. To the best of our knowledge there is no study conducted on the comparative analysis of EPs extraction methods from colostrum.

In our study we thoroughly studied various extraction methods for the efficient extraction of EPs from colostrum. Although all the methods identified more than thousands peptide sequences but TCA and MWCO identified highest number of peptide sequences. Comparatively less number of identification in LL extraction can be the co-precipitation of peptides (Fig. 2A). Dingess and coworkers analysed three different methods to identify endogenous peptides from human milk which were TCA precipitation, MWCO and LL extraction and were able to identify maximum of 3237 peptides using TCA precipitation. Our study aims to develop an efficient method for extraction of EPs from bovine colostrum which has different chemical composition than human milk and in the present study we identified 3211 peptides using TCA precipitation and 3210 unique peptides using MWCO method. Peptides identified using MWCO methods were higher in number than previous studies where this same method was used to identify EPs. The less number of identification using the molecular weight cutoff method in previous studies could be due to clogging of the filter membrane which occurs due to the high molecular weight proteins present in the colostrum which hinders the subsequent filtration of peptides through the filter membrane. Also, a study speculated the hindrance in peptide extraction using 10 kDa filters can be due to adsorption of peptides on filter membrane *via* electrostatic interaction.^46^ Therefore, we used ACN in combination with MWCO for extraction of EPs. ACN helps to dissociate the interaction of low abundant proteins and peptides which remain bound to the carrier proteins so these small and low abundant proteins and peptides become available to be detected by LC-MS/MS.^3^ Additionally, this huge number of exclusive identification may stem from a single amino acid residue difference at both the N and C-termini. Another reason for this could be the diversification of peptide sequences across methods which may contribute to this large number. Notably, heat treatment is implicated in inducing chemical reactions such as Maillard reactions and oxidation, leading to the emergence of post-translationally modified sequences.^12^

Our objective was to isolate low molecular weight peptides, a goal successfully realized through each employed EPs extraction method. All methods demonstrated a predominant presence of peptides within the mass range of 700–2000 Da and amino acid residues in sequences between 7 and 20 amino acids mostly (Fig. 3C and D). Furthermore, our study aligns with prior research focused on extracting low molecular weight peptides, confirming the consistency of our findings with existing literature.^23,41^

Parent proteins analysis of identified sequences revealed that some major proteins were consistently enriched through all the methods including beta casein, alpha S1 casein, kappa casein, GlyCAM, WASH complex, WASL, polymeric immunoglobulin receptor, whirlin protein, histone lysine-*N*-methyltransferase, proline rich coiled coil and some uncharacterized proteins (Fig. 4). It was noteworthy that no high confidence peptide was obtained from alpha S2 in TCA, heat treated and LL extraction methods, although peptides were obtained from Alpha S2 through MWCO method. Beta lactoglobulin protein contributed peptides in TCA, MWCO and heat treated method but no peptide was observed in LL extracted method. In line with the previous studies, no EPs were observed from alpha lactalbumin.^17^ Other major proteins such as perilipin and butyrophilin, reported in prior research, yielded few high-confidence peptides in our study. Peptides from butyrophilin were exclusively observed in the heat-treated method and peptide originating from perilipin were identified in MWCO and HT methods only. It is noteworthy that EPs from proteins such as whirlin, WASH complex, and WASL were not previously reported in the milk or colostrum of any species, expanding our understanding of the peptide composition in these protein complexes.

An examination of the GRAVY index, molecular weight, amino acid length, and other physico-chemical properties of the identified peptides revealed that all methods enriched comparable peptide types. This indicates no discernible distinctions in the chemical–physical attributes of the identified peptides (Fig. 3A–D).

Functional annotation of the sequences indicated their potential bioactivity. All methods successfully extracted peptides previously obtained from cow or yak milk or colostrum including those with demonstrated antihypertensive, antioxidative, anti-inflammatory, and immunomodulatory effects (Table 1). Although, there were slight variations in the N or C-terminus or sequence length of these peptides across different extraction methods as shown in Table 1, all similarly extracted peptides exhibited proven bioactivity.

Some key endogenous peptides have been previously isolated from colostrum. For instance, casecidin 15 (YQEPVLGPVRGPFPI) and casecidin 17 (YQEPVLGPVRGPFPIIV) from bovine colostrum have demonstrated antimicrobial properties. Another peptide, isracidine (RPKHPIKHQGLPQEVLNENLLRF), also shows antimicrobial activity and has been isolated from bovine colostrum.^34^ In the present study, either the full-length sequence or a fragment within these sequences (casecidin 15 or casecidin 17 or isracidine) was isolated using all methods. Specifically, casecidin 17 was isolated through MWCO, HT, and liquid–liquid extraction, but not through TCA. However, fragments of this peptide was present in the TCA based extraction method, which also has proven biological activities. Additionally, the fragment YQEPVLGPVR, found within the sequences of casecidin 15 and 17, was observed only through the MWCO method. Previous studies have demonstrated its anti-inflammatory, antioxidative, and immunomodulatory potential.^47^ Furthermore, another peptide, KVLPVPQ, with proven ACE-inhibitory, immunomodulatory, and anti-inflammatory properties,^48,49^ was consistently isolated through all extraction methods. Overall, most methods were successful in isolating peptides with bioactive potential, with the MWCO method being particularly effective in extracting the exact sequence of casecidin 17 and its other fragments having proven biological activities. Therefore, this study confirms the bioactive potential of peptides extracted from colostrum, with consistent results across different extraction methods despite slight variations in peptide sequences.

These findings collectively underscore the potential bioactivity inherent in the identified sequences across employed methods.

Given the similarity in the physicochemical characteristics—such as amino acid profile, sequence length, and molecular weight—of the peptides identified *via* different extraction methods, it is challenging to determine the most effective extraction technique for extraction of EPs from colostrum. The bioactive potential of the peptides, assessed through PeptideRanker and validated using MBPDB, further complicates direct comparison of the efficacy of these methods since all the method successfully extracted peptides with bioactive peptides.

To evaluate the efficiency of the applied extraction techniques, we considered two primary metrics: the number of identified sequences and the number of proteins covered by each method for extracting EPs. Our findings revealed that TCA precipitation and MWCO methods yielded the highest number of peptides, derived from a broad range of parent proteins, particularly from low abundant proteins. Conversely, the other two methods evaluated demonstrated lower peptide yields. These results underscore the importance of employing TCA precipitation or MWCO methods. Thus, we focused our comparative analysis on TCA and MWCO methods. Several measures were taken into consideration to assess efficiency of these methods (Table S1†): 1. Number of peptides identified and number of unique peptides in both methods, 2. Percentage peak area occupied by total peptides, shared peptides, and key peptides reported in literature, 3. Number of parent proteins, 4. Percentage of peptides scoring more than 0.5 or more than 0.1 in PeptideRanker, 5. Number of key endogenous peptides identified, 6. Amino acid composition and 7. The major proteins from which the EPs have already been isolated from milk and colostrum.

The number of peptides identified was nearly equal for both methods. However, TCA identified a higher number of unique peptides, as shown in Fig. 2B. Additionally, TCA precipitation method resulted in a higher percentage peak area for total identified peptides, peptides shared by both methods, and the number of parent proteins contributing to the extraction of EPs.

Sequence logos analysis of the first seven amino acids from the N-terminus revealed that leucine and proline were the most dominant amino acids in nearly all positions for both methods. Despite these strengths, the bioactive functionality analysis showed that MWCO identified more bioactive sequences compared to TCA. Specifically, 27.25% of sequences identified by MWCO had a PeptideRanker score of 0.5 or higher, compared to 26.41% for TCA. Furthermore, 20 peptides identified through MWCO displayed bioactive potential when screened through the MBPDB, compared to 13 peptides for TCA.

Moreover, the percentage peak area occupied by key EPs identified by both methods was higher in the MWCO method. Another interesting metric advocating the use of the MWCO method is the observation that major proteins, such as perilipin and alpha S2 casein, yielded EPs only in MWCO and not in TCA.

Therefore, while the TCA precipitation method excels in identifying a large number of sequences and parent proteins, the MWCO method is superior for isolating bioactive EPs from almost all the major proteins and EPs suitable for therapeutic screening.

## Conclusions

This research addresses the development of an efficient extraction procedure from the colostrum matrix, evaluating and comparing the efficacy of various methods for extracting EPs from colostrum. While previous studies have demonstrated EPs extraction from milk, serum, and other biological fluids, to our knowledge, no study has compared the extraction efficiency of methods specifically on colostrum. The novelty of this study lies in the extraction of bioactive EPs from colostrum using peptide extraction methods combined with ultracentrifugation to deplete the most abundant proteins (casein). Using ultracentrifugation in combination with the existing methods with some modifications enables the identification of less abundant proteins and peptides. In conclusion, we recommend the use of MWCO for the identification of peptides with bioactive properties due to its comparatively wider coverage of bioactive peptides in available database. However, the TCA method remains valuable for quantitative purposes and for identifying a high number of peptides from diverse parent proteins. Both methods have their unique strengths and can be chosen based on the specific goals of the research, ensuring a comprehensive approach to peptide extraction and analysis. Through our detailed evaluation and selection process, we have achieved the purpose of our research by identifying the most suitable technique for peptide extraction based on the desired outcomes. This work significantly contributes to bridging the gap in developing efficient extraction procedures for the colostrum matrix.

## Data availability

The mass spectrometry proteomics data have been deposited with the ProteomeXchange consortium *via* the PRIDE^50^ partner repository with the dataset identifier PXD051733 (PRIDE reference: 1-20240425-095929-3277975).

## Author contributions

P. P.: conceptualization, design and conduct of experiments, data analysis, interpretation of results, investigation, data visualization, writing – original draft preparation, review and editing; R. R.: writing – original draft preparation, review and editing; R. K.: design of experiments, writing – review and editing; S. M.: writing – review and editing; S. K.: sample collection, conceptualization, supervision, data analysis, writing – review and editing; M. M.: sample collecion, suggestion in design of experiment, writing – review and editing, J. K. K., M. S. and A. K. M.: suggestion in design of experiment, writing – review and editing. All authors read and approved the final manuscript.

## Conflicts of interest

There are no conflicts to declare.

## Supplementary Material

RA-014-D4RA03199G-s001

## References

[cit1] Aristoteli L. P., Molloy M. P., Baker M. S. (2007). Evaluation of endogenous plasma peptide extraction methods for mass spectrometric biomarker discovery. J. Proteome Res..

[cit2] Ferranti P., Traisci M. V., Picariello G., Nasi A., Boschi V., Siervo M., Falconi C., Chianese L., Addeo F. (2004). Casein proteolysis in human milk: tracing the pattern of casein breakdown and the formation of potential bioactive peptides. J. Dairy Res..

[cit3] Merrell K., Southwick K., Graves S. W., Esplin M. S., Lewis N. E., Thulin C. D. (2004). Analysis of low-abundance, low-molecular-weight serum proteins using mass spectrometry. J. Biomol. Tech..

[cit4] Kim B. J., Dallas D. C. (2021). Systematic examination of protein extraction, proteolytic glycopeptide enrichment and MS/MS fragmentation techniques for site-specific profiling of human milk N-glycoproteins. Talanta.

[cit5] Piovesana S., Cerrato A., Antonelli M., Benedetti B., Capriotti A. L., Cavaliere C., Montone C. M., Laganà A. (2020). A clean-up strategy for identification of circulating endogenous short peptides in human plasma by zwitterionic hydrophilic liquid chromatography and untargeted peptidomics identification. J. Chromatogr. A.

[cit6] Kumar R., Ali S. A., Singh S. K., Bhushan V., Kaushik J. K., Mohanty A. K., Kumar S. (2021). Peptide profiling in cow urine reveals molecular signature of physiology-driven pathways and in-silico predicted bioactive properties. Sci. Rep..

[cit7] Jørgensen A. L., Juul-Madsen H. R., Stagsted J. (2010). Colostrum and bioactive, colostral peptides differentially modulate the innate immune response of intestinal epithelial cells. J. Pept. Sci..

[cit8] Dallas D. C., Weinborn V., de Moura Bell J. M., Wang M., Parker E. A., Guerrero A., Hettinga K. A., Lebrilla C. B., German J. B., Barile D. (2014). Comprehensive peptidomic and glycomic evaluation reveals that sweet whey permeate from colostrum is a source of milk protein-derived peptides and oligosaccharides. Food Res. Int..

[cit9] Montone C. M., Capriotti A. L., Cerrato A., Antonelli M., La Barbera G., Piovesana S., Laganà A., Cavaliere C. (2019). Identification of bioactive short peptides in cow milk by high-performance liquid chromatography on C18 and porous graphitic carbon coupled to high-resolution mass spectrometry. Anal. Bioanal. Chem..

[cit10] Zhang L., Han B., Luo B., Ni Y., Bansal N., Zhou P. (2022). Characterization of endogenous peptides from dromedary and bactrian camel milk. Eur. Food Res. Technol..

[cit11] Montone C. M., Aita S. E., Cavaliere C., Cerrato A., Laganà A., Piovesana S., Capriotti A. L. (2021). High-resolution mass spectrometry and chemometrics for the detailed characterization of short endogenous peptides in milk by-products. Molecules.

[cit12] Wölk M., Milkovska-Stamenova S., Hoffmann R. (2020). Comprehensive profiling of the native and modified peptidomes of raw bovine milk and processed milk products. Foods.

[cit13] Zhu J., Dingess K. A., Mank M., Stahl B., Heck A. J. (2021). Personalized profiling reveals donor-and lactation-specific trends in the human milk proteome and peptidome. J. Nutr..

[cit14] Wang X., Sun Y., Wang F., You L., Cao Y., Tang R., Wen J., Cui X. (2020). A novel endogenous antimicrobial peptide CAMP 211-225 derived from casein in human milk. Food Funct..

[cit15] Enjapoori A. K., Kukuljan S., Dwyer K. M., Sharp J. A. (2019). In vivo endogenous proteolysis yielding beta-casein derived bioactive beta-casomorphin peptides in human breast milk for infant nutrition. Nutrition.

[cit16] Dingess K. A., Gazi I., van den Toorn H. W., Mank M., Stahl B., Reiding K. R., Heck A. J. (2021). Monitoring human milk β-casein phosphorylation and O-glycosylation over lactation reveals distinct differences between the proteome and endogenous Peptidome. Int. J. Mol. Sci..

[cit17] Dallas D. C., Guerrero A., Parker E. A., Garay L. A., Bhandari A., Lebrilla C. B., Barile D., German J. B. (2014). Peptidomic profile of milk of Holstein cows at peak lactation. J. Agric. Food Chem..

[cit18] Bhattacharya M., Salcedo J., Robinson R. C., Henrick B. M., Barile D. (2019). Peptidomic and glycomic profiling of commercial dairy products: identification, quantification and potential bioactivities. NPJ Sci. Food.

[cit19] Dallas D. C., Guerrero A., Khaldi N., Castillo P. A., Martin W. F., Smilowitz J. T., Bevins C. L., Barile D., German J. B., Lebrilla C. B. (2013). Extensive *in vivo* human milk peptidomics reveals specific proteolysis yielding protective antimicrobial peptides. J. Proteome Res..

[cit20] Nielsen S. D., Beverly R. L., Dallas D. C. (2017). Peptides released from foremilk and hindmilk proteins by breast milk proteases are highly similar. Front. Nutr..

[cit21] Guerrero A., Dallas D. C., Contreras S., Bhandari A., Cánovas A., Islas-Trejo A., Medrano J. F., Parker E. A., Wang M., Hettinga K., Chee S. (2015). Peptidomic analysis of healthy and subclinically mastitic bovine milk. Int. Dairy J..

[cit22] Yu W., Yu Y., Wang W., Li Y., Szeto I. M., Jin Y. (2021). Effect of sample preparation on analysis of human milk endogenous peptides using liquid chromatography-tandem mass spectrometry. Sepu.

[cit23] Piovesana S., Capriotti A. L., Cavaliere C., La Barbera G., Samperi R., Chiozzi R. Z., Laganà A. (2015). Peptidome characterization and bioactivity analysis of donkey milk. J. Proteom..

[cit24] Nissen A., Andersen P. H., Bendixen E., Ingvartsen K. L., Røntved C. M. (2017). Colostrum and milk protein rankings and ratios of importance to neonatal calf health using a proteomics approach. J. Dairy Sci..

[cit25] Abdel-Hamid M., Yang P., Mostafa I., Osman A., Romeih E., Yang Y., Huang Z., Awad A. A., Li L. (2022). Changes in whey proteome between Mediterranean and Murrah buffalo colostrum and mature milk reflect their pharmaceutical and medicinal value. Molecules.

[cit26] Kashyap R., Narayan K. S., Vij S. (2022). Identification of antibacterial and immunomodulatory bioactive peptides generated from buffalo (Bubalus bubalis) colostrum whey fermented by Lactobacillus rhamnosus C25: LC-MS/MS-based analysis. J. Funct. Foods.

[cit27] Fajardo-Espinoza F. S., Ordaz-Pichardo C., Sankar U., Romero-Rojas A., Moreno-Eutimio M. A., Hernández-Sánchez H. (2021). In vitro cytomodulatory and immunomodulatory effects of bovine colostrum whey protein hydrolysates. Int. J. Food Sci. Technol..

[cit28] Chae A., Aitchison A., Day A. S., Keenan J. I. (2017). Bovine colostrum demonstrates anti-inflammatory and antibacterial activity in *in vitro* models of intestinal inflammation and infection. J. Funct. Foods.

[cit29] Kh T. K. (2023). Peptides of trypsin hydrolyzate in bovine colostrum. Food Process. Tech. Technol..

[cit30] Ashok N. R., Vivek K. H., Aparna H. S. (2019). Antioxidative role of buffalo (Bubalus bubalis) colostrum whey derived peptides during oxidative damage. Int. J. Pept. Res. Ther..

[cit31] Madsen B. D., Rasmussen M. D., Nielsen M. O., Wiking L., Larsen L. B. (2004). Physical properties of mammary secretions in relation to chemical changes during transition from colostrum to milk. J. Dairy Res..

[cit32] Dziewiecka H., Buttar H. S., Kasperska A., Ostapiuk-Karolczuk J., Domagalska M., Cichoń J., Skarpańska-Stejnborn A. (2022). A systematic review of the influence of bovine colostrum supplementation on leaky gut syndrome in athletes: diagnostic biomarkers and future directions. Nutrients.

[cit33] Campanhon I. B., de Aguiar P. F., Bezerra F. F., Soares M. R., Torres A. G. (2023). Human colostrum *in vitro* protein digestion: peptidomics by LC-Orbitrap-HRMS and prospection for bioactive peptides *via* bioinformatics. Br. J. Nutr..

[cit34] Birkemo G. A., O’sullivan O., Ross R. P., Hill C. (2009). Antimicrobial activity of two peptides casecidin 15 and 17, found naturally in bovine colostrum. J. Appl. Microbiol..

[cit35] Zhou Y., Zhang L., Yu Z., Zhang A., Wu W., Chen W., Yan X., Liu H., Hu Y., Jiang C., Xu Y. (2019). Peptidomic analysis reveals multiple protection of human breast milk on infants during different stages. J. Cell. Physiol..

[cit36] Cui X., Li Y., Yang L., You L., Wang X., Shi C., Ji C., Guo X. (2016). Peptidome analysis of human milk from women delivering macrosomic fetuses reveals multiple means of protection for infants. Oncotarget.

[cit37] Oliveros J. C. , VENNY. An interactive tool for comparing lists with Venn Diagrams. 2007. https://bioinfogp.cnb.csic.es/tools/venny/index.html

[cit38] Tang D., Chen M., Huang X., Zhang G., Zeng L., Zhang G., Wu S., Wang Y. (2023). SRplot: a free online platform for data visualization and graphing. PLoS ONE.

[cit39] Spitzer M., Wildenhain J., Rappsilber J., Tyers M. (2014). BoxPlotR: a web tool for generation of box plots. Nat. Methods.

[cit40] Sharma A., Kapoor P., Gautam A., Chaudhary K., Kumar R., Chauhan J. S., Tyagi A., Raghava G. P. (2013). Computational approach for designing tumor homing peptides. Sci. Rep..

[cit41] Dingess K. A., van den Toorn H. W., Mank M., Stahl B., Heck A. J. (2019). Toward an efficient workflow for the analysis of the human milk peptidome. Anal. Bioanal. Chem..

[cit42] Guerrero A., Dallas D. C., Contreras S., Chee S., Parker E. A., Sun X., Dimapasoc L., Barile D., German J. B., Lebrilla C. B. (2014). Mechanistic peptidomics: factors that dictate specificity in the formation of endogenous peptides in human milk. Mol. Cell. Proteom..

[cit43] Ning J., Yang M., Zhu Q., Luo X., Yue X. (2024). Peptidomics comparison of endogenous peptides derived from parent proteins in bovine colostrum and mature milk. LWT.

[cit44] Nissen A., Bendixen E., Ingvartsen K. L., Røntved C. M. (2012). In-depth analysis of low abundant proteins in bovine colostrum using different fractionation techniques. Proteomics.

[cit45] Peng J., Zhang H., Niu H. (2020). Peptidomic analyses: the progress in enrichment and identification of endogenous peptides. TrAC, Trends Anal. Chem..

[cit46] Du Y., Wu D., Wu Q., Guan Y. (2015). Quantitative evaluation of peptide-extraction methods by HPLC-triple-quad MS-MS. Anal. Bioanal. Chem..

[cit47] Sowmya K., Bhat M. I., Bajaj R. K., Kapila S., Kapila R. (2019). Buffalo milk casein derived decapeptide (YQEPVLGPVR) having bifunctional anti-inflammatory and antioxidative features under cellular milieu. Int. J. Pept. Res. Ther..

[cit48] Adams C., Sawh F., Green-Johnson J. M., Taggart H. J., Strap J. L. (2020). Characterization of casein-derived peptide bioactivity: differential effects on angiotensin-converting enzyme inhibition and cytokine and nitric oxide production. J. Dairy Sci..

[cit49] Maeno M., Yamamoto N., Takano T. (1996). Identification of an antihypertensive peptide from casein hydrolysate produced by a proteinase from Lactobacillus helveticus CP790. J. Dairy Sci..

[cit50] Perez-Riverol Y., Bai J., Bandla C., García-Seisdedos D., Hewapathirana S., Kamatchinathan S., Kundu D. J., Prakash A., Frericks-Zipper A., Eisenacher M., Walzer M. (2022). The PRIDE database resources in 2022: a hub for mass spectrometry-based proteomics evidences. Nucleic Acids Res..

