# Supplementary material for: Optimizing workflow efficiency for analyzing low molecular weight endogenous peptides in colostrum
Source: RSC Adv. 2024 Sep 16;14(40):29189–200. doi: 10.1039/d4ra03199g (PMC11403347; doi:10.1039/d4ra03199g)
Supplement: RA-014-D4RA03199G-s001 [file RA-014-D4RA03199G-s001.pdf]

Supplementary Information (SI) for RSC Advances.
This journal is © The Royal Society of Chemistry 2024

Supplementary Table S1: Different criteria used to evaluate the efficiency of TCA and MWCO extraction methods:

**S.No.**	**criteria**	**TCA**	**MWCO**
**1**	**Number of peptides identified**	3211	3210
**2**	**Number of unique peptides**	2942	2884
**3**	**Percentage peak area occupied by total peptides**	55.12%	41.44%
**4**	**Percentage peak area occupied by shared peptides**	15.44%	13.57%
**5**	**Percentage peak area of key peptides**	0.9%	1.88%
**6**	**Number of parent protein**	2647	2587
**7**	**Percentage of peptides having score more than 0.5 in PeptideRanker**	26.41%	27.25%
**8**	**Percentage of peptides having score more than 0.1 in PeptideRanker**	89.44%	89.46%
**9**	**Number of key EPs identified**	13	20
**10**	**Amino acid at 1st position**	K>L>Q	L>K>P
	**2nd position**	L>P>A	P>L>V
	**3rd position**	L>P>S	L>P>V
	**4TH position**	L>P>A	L>P>A
	**5th position**	L>P>S	L>P>G

Supplementary Table S2: Percentage peak area occupied by some representative EPs extracted via TCA precipitation and MWCO methods:

**S.No.**	**Peptide reported in literature**	**Peak area occupied (%)**	
		**TCA**	**MWCO**
1	EPVLGPVRGP	0.1052	0.1178
2	VLGPVRGPFP	0.0300	0.1670
3	EPVLGPVRGPFP	0.5559	1.5174
4	SQSKVLPVPQKAVPYPQ	0.0203	0.0092
5	TPVVVPPFL	0.0043	0.0144
6	KVLPVPQ	0.0477	0.0379
7	RPKHPIKHQ	0.1369	0.015
8	KVLPVPQK	0.0136	0.0111
